# Adipose Stromal Cell Expansion and Exhaustion: Mechanisms and Consequences

**DOI:** 10.3390/cells9040863

**Published:** 2020-04-02

**Authors:** Kristin Eckel-Mahan, Aleix Ribas Latre, Mikhail G. Kolonin

**Affiliations:** 1 Institute of Molecular Medicine, McGovern Medical School at the University of Texas Health Science Center, Houston, TX 77030, USA; Kristin.L.Mahan@uth.tmc.edu; 2 Helmholtz Institute for Metabolic, Obesity and Vascular Research Center, D-04103 Leipzig, Germany; Aleix.RibasLatre@uth.tmc.edu

**Keywords:** adipose tissue, brown adipocytes, stromal cells, adipocyte progenitors, circadian clock, proliferation, hypertrophy, hyperplasia, senescence, obesity, diabetes

## Abstract

Adipose tissue (AT) is comprised of a diverse number of cell types, including adipocytes, stromal cells, endothelial cells, and infiltrating leukocytes. Adipose stromal cells (ASCs) are a mixed population containing adipose progenitor cells (APCs) as well as fibro-inflammatory precursors and cells supporting the vasculature. There is growing evidence that the ability of ASCs to renew and undergo adipogenesis into new, healthy adipocytes is a hallmark of healthy fat, preventing disease-inducing adipocyte hypertrophy and the spillover of lipids into other organs, such as the liver and muscles. However, there is building evidence indicating that the ability for ASCs to self-renew is not infinite. With rates of ASC proliferation and adipogenesis tightly controlled by diet and the circadian clock, the capacity to maintain healthy AT via the generation of new, healthy adipocytes appears to be tightly regulated. Here, we review the contributions of ASCs to the maintenance of distinct adipocyte pools as well as pathogenic fibroblasts in cancer and fibrosis. We also discuss aging and diet-induced obesity as factors that might lead to ASC senescence, and the consequences for metabolic health.

## 1. Introduction

**Figure 1:**
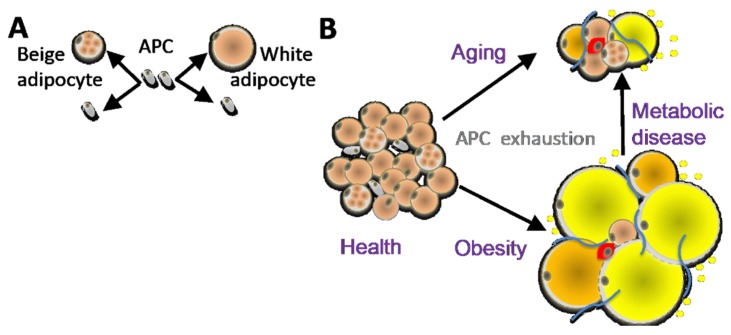
(**A**) Distinct adipose progenitor cell (APC) lineages maintain pools of white and beige adipocytes. (**B**) In aging and obesity, APC senescence results in adipocyte, hypertrophy, hypoxia and death, causing leukocyte infiltration (red), inflammation, fibrosis (blue), and lipid incontinence (yellow).

Adipose tissue (AT) is a highly dynamic organ that mediates glucose and lipid homeostasis. The expansion of white adipose tissue (WAT) is a hallmark of obesity, and visceral adipose tissue (VAT) dysfunction, in particular, is thought to help fuel metabolic syndromes, type-2 diabetes (T2D), cardiovascular disease, and cancer [1]. In contrast, healthy subcutaneous adipose tissue (SAT) can display beneficial properties, mediated in part by its ability to take up and catabolize excess circulating lipids [2]. Adipose tissue is comprised of a diverse number of cell types, including adipocytes, stromal cells, endothelial cells and infiltrating leukocytes [1]. Adipose stromal cells (ASCs) are a mixed population containing adipose progenitor cells (APCs), as well as cells that support angiogenesis and vascular integrity [3]. Adipocytes, the differentiated lipid-rich cells and the main constituents of AT, are also heterogeneous and have depot-specific ontogeny [4]. In adults, large AT depots are comprised of predominantly white, triglyceride-storing adipocytes with unilocular lipid droplets. While the lipid droplet size of white adipocytes increases as a result of adipogenesis/lipogenesis and fatty acid (FA) uptake, it decreases in response to the sympathetic nervous system (SNS) activation of β-adrenergic receptor by catecholamines [5]. While VAT shows a weaker response to SNS stimuli, β-adrenergic signaling in SAT can activate lipolysis [6,7], mitochondrial biogenesis, and non-shivering adaptive thermogenesis [8,9,10]. In mice and humans, SAT contains inducible brown-like (beige aka brite) adipocyte [5,11]. These mitochondria-rich adipocytes with high lipolytic activity can be recruited, and they are functionally similar to brown adipocytes in canonical brown adipose tissue (BAT) [12,13]. Uniquely, brown and beige adipocytes comprise the ability to burn FA and dissipate energy as heat. They also contribute to whole body glycemia by metabolizing glucose (reviewed in [14]). This function depends on the activity of uncoupling protein 1 (UCP1), which leaks protons to uncouple substrate oxidation from ATP synthesis [15]. In contrast to BAT, WAT is largely devoid of UCP1 expression. Due to high metabolic activity, BAT and beige AT can help counteract hyperglycemia and some of the other metabolic consequences of obesity [2,16]. The presence of UCP1 and its activity in the beige AT of adult humans has been demonstrated [2,17,18]. Healthy metabolism relies on the ability of AT to uptake lipids, preventing excess fat accumulation in other tissues such as the liver, skeletal muscle, and the pancreas at times of positive energy balance [19]. The presence of thermogenic beige adipocytes and lipid-storing white adipocytes in AT underlie this metabolic activity [5]. Metabolic effects that have been observed in animal models reveal that the activation of beige adipocytes correlates with the suppression of insulin resistance (IR) and type-2 diabetes development in obesity [20]. However, even without thermogenesis, healthy AT composed of small and well-vascularized adipocytes can protect against steatosis and lipotoxicity contributing to metabolic disease [21,22] (Figure 1).

**Figure 2:**
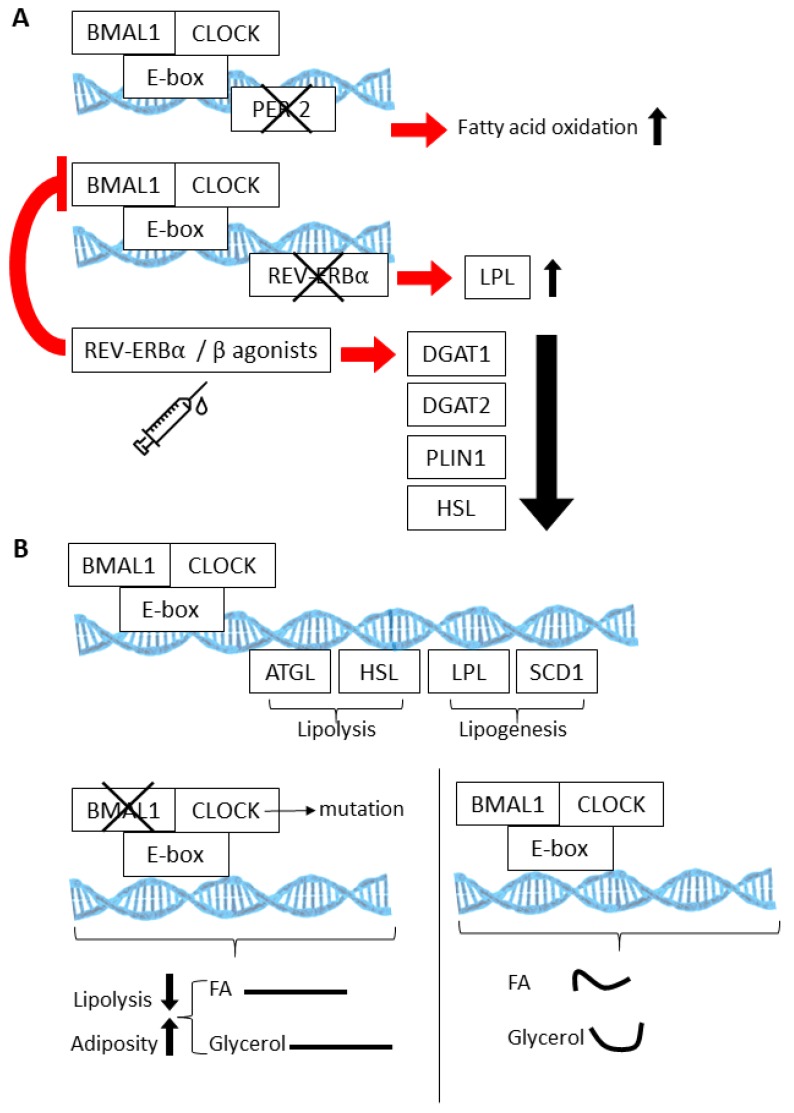
The BMAL1:CLOCK (Aryl Hydrocarbon Receptor Nuclear Translocator Like (AHRNTL aka BMAL):(Circadian Locomotor Output Cycles Protein Kaput)) heterodimer regulates multiple genes involved in fatty acid oxidation (**A**), lipogenesis, and lipolysis (**B**). Knockout models involving loss of the circadian proteins PER2, REV-ERBA, and BMAL1 have elucidated the physiological consequences of these activities within the context of white adipose tissue (WAT) and brown adipose tissue (BAT) (also see Table 1). (Curved lines are indicative of rhythmic release.).

AT is an organ that is under constant remodeling and that accommodates fluctuations in energy availability, in part by dynamic fluctuations in lipolysis and fatty acid oxidation (Figure 2) [23,24]. With excessive energy, AT expansion relies on the proliferation of APCs to generate new adipocytes [12]. In addition, the expansion of other stromal cells (such as pericytes) and vascular endothelial cells ensures sufficient blood flow and modulates the remodeling process by compensating for cell loss from cell death and degenerative conditions [25]. Blood flow in AT is highly labile and alters in response to external factors (fasting, postprandial, and exercise) [26]. Nevertheless, AT is described to be a relatively hypoxic tissue that receives about 5% of total cardiac output [27].Therefore, the rapid expansion of WAT may further exacerbate the hypoxic condition, in particular in morbid obesity featuring extreme adipocyte enlargement. ASCs are activated under ischemic conditions and are fundamental to AT remodeling and expansion [28]. Adipocytes are continuously replaced throughout aging, and their pools in fat tissue are maintained by APCs, which divide to self-renew and differentiate (Figure 1A). The turnover rate of adipocytes and pre-adipocytes has been debated, though some studies have suggested a 10% yearly turnover of adipocytes in adult humans [29]. This number changes under conditions of nutrient challenge that cause WAT expansion, and it is likely also depot-specific [29,30]. The turnover of adipocytes and pre-adipocytes in mice is higher than in humans [21,31]. It has been determined that 1%–1.5% of pre-adipocytes undergo replication per day [32], which is consistent with more than 1% of adipocytes undergoing turnover every day according to another study [33]. A growing body of evidence suggests that sustained proliferative and adipogenic APC competence, which supports AT maintenance through adipocyte hyperplasia rather than hypertrophy, explains the lack of T2D development in “healthy obese” individuals [21,22]. Adipocyte hypertrophy leads to hypoxia and cell death, resulting in the recruitment of inflammatory leukocytes [34]. When this chronic process becomes chronic in obesity or aging, it results in inflammation, fibrosis, and lipid spillover (Figure 1B). A better understanding of how APC capacity to generate new adipocytes is regulated is essential for the development of new approaches for the prevention of metabolic disease.

## 2. Origins and Functions of Adipocyte Progenitors within Fat Tissue

The conversion of beige fat to white or vice versa during development or in response to physiological cues is not completely understood. Lineage tracing data have provided evidence for both the trans-differentiation of existing adipocytes [35] and the recruitment of new APCs in this process [11,36,37,38]. The proliferation and differentiation of APCs and their subsequent development into hyperplastic adipocytes underlies AT remodeling in conditions of excess energy [1,39,40]. We and others have shown that APCs are subpopulations of ASCs, the fibroblastic cells of the stromal/vascular cell (SVC) fraction [41,42,43]. APCs can undergo differentiation into distinct lineages of preadipocytes, depending on developmental and environmental cues [44,45,46,47], with distinct subpopulations of APCs having propensity for beige vs. white adipogenesis [36,48,49] (Figure 1A).

The cells of WAT can be separated by enzymatic digestion into a buoyant fraction and a pelleted stromal vascular fraction (SVF) [23,50,51]. The buoyant fraction is enriched with lipid-laden pre-adipocytes and mature adipocytes, whereas the pelleted SVF is constituted by a heterogeneous population of cells: adipose progenitor cells/stromal cells (ASCs), adipose endothelial cells (AECs), and infiltrating hematopoietic cells [23]. To date, there has been no definitive description of the immunophenotypic characteristic of ASCs [50]. Our group and others have utilized their plastic adherence properties and CD34-positive CD45-negative CD31-negative (CD34+CD45-CD31-) immunophenotype to isolate and enrich for ASCs [3]. Like mesenchymal stromal cells (MSCs) in bone marrow, APCs are marked by membrane tyrosine kinase platelet-derived growth factor receptors alpha (PDGFRα) and beta (PDGFRβ) [3]. In vivo, most APCs express PDGFRα, while PDGFRβ is expressed only in some APCs [52,53]. It has been shown that the *Pdgfrα+* lineage contributes to all adipocytes in SAT [48,53], while the *Pdgfrβ+* lineage contributes to subsets of white and beige adipocytes [40,41]. While the constitutive activation of PDGFRα induces fibrosis [45,54], its transient activation induces AT beiging [55]. We reported that a compound targeting PDGFRβ+ ASCs but sparing PDGFRα+ ASCs induces AT beiging in mice [52]. This suggested that beige adipocytes are derived from PDGFRα+/PDGFRβ-APC in adulthood. Supporting this notion, our previous lineage tracing study [56] demonstrated that *Pdgfrα* expression precedes *Pdgfrβ* expression in almost all subcutaneous but only in a fraction of visceral ASCs, indicating two distinct APC lineages in VAT. We showed that HFD feeding or thermoneutrality induces *Pdgfrβ* lineage recruitment to predominantly generate white adipocytes in SAT and VAT, while it is the *Pdgfrα* lineage that is primed to generate beige adipocytes in VAT [56]. PDGFR activity is regulated by PDGFs, the ligands that function as dimers [57]. PDGF-AA is a selective activator of PDGFRα, while PDGF-DD is a selective activator of PDGFRβ. We showed that PDGF-AA induces AT beiging, while PDGF-DD induces AT whitening [56]. This report concluded that the balance of transient PDGFRα/PDGFRβ expression and signaling during adipogenesis induction defines whether preadipocytes differentiate as beige or white, respectively.

## 3. Regulation of Adipocyte Progenitor Cell Proliferation

The ability of APCs to proliferate is a critical component of healthy AT and in contrast to what occurs in unhealthy fat where already existing adipocytes become hypertrophic and macrophage accumulation and fibrosis occurs [58]. While mature adipocytes are thought to be limited in their ability to proliferate, APCs can undergo robust and rapid proliferation, which is dependent on a number of factors, including lineage specification and energy surplus or depletion. WAT is known to be innervated by sympathetic neurons (reviewed in [59]), and APC proliferation is highly influenced by β-adrenergic signaling, though diet also plays a significant role [53]. Even eight weeks of HFD feeding in rodents can increase the proliferative capacity of PDGFRα-positive progenitor cells by over 12-fold in visceral fat. There is also evidence that WAT expansion in response to such a dietary challenge is highly depot-specific. For example, while HFD produces VAT expansion via both hypertrophy and hyperplasia, SAT expansion predominantly occurs as a result of hypertrophy [11,60]. In VAT, hyperplasia in response to HFD has also been shown to be due, in part, to *Pdgfrb*-positive perivascular precursor cells, which express high levels of the Zinc Finger Protein 423 (*Zfp423*) and show a high commitment for generating preadipocytes [40].

Though the characterization of adipocyte progenitors has traditionally relied on their identification by using flow cytometry based on antibodies against cell surface markers, recent studies that have used single-cell RNA sequencing have greatly expanded our understanding of the lineage-specific origins of new adipocytes in fat tissue [47]. For example, within the pool of *Pdgfrb*-positive cells in VAT, single-cell RNA sequencing has revealed substantial heterogeneity, with cells falling into several clusters: 1) adipocyte precursor cells, 2) committed preadipocytes, 3) “fibro-inflammatory” progenitors, and 4) “mesothelial-like” cells [47]. While a prominent cluster shows a greater propensity for fibrotic and inflammatory phenotypes, others (LY6C-CD9-PDGFRB+) represent a distinct visceral APC population that can spontaneously differentiate in vitro in response to insulin-containing media [47]. In SAT, interstitial progenitor cells expressing dipeptidyl peptidase-4 (DPP4+) are highly proliferative and resist an adipogenic cell fate, serving instead as multipotent progenitor cells. During SAT development, a fraction of DPP4-positive cells ultimately give rise to two classes of adhesion molecule-1-expressing (ICAM1+) cells, one of which is a preadipocyte and the other being an adipogenic population of cells identified by the unique co-expression of C-Type Lectin Domain Containing 11ª (*Clec11aII*) and Coagulation Factor III (F3/*CD142*). Thus far, it appears that these unique populations of mesenchymal cells are present across several different AT depots and under various nutrient conditions [47,61]. While this distinct hierarchy of adipose progenitors has only been recently defined, it will be interesting to further elucidate what drivers induce DPP4-positive cells to adopt an adipogenic cell fate vs. a proliferative one. In a study by Merrick et al. [61], transforming growth factor beta (TGFβ) signaling appeared to be a central mechanism by which proliferation over differentiation is achieved. However, ICAM1+ preadipocytes become resistant to TGFβ’s proliferative effects.

In regards to AT development, the number of adipocytes in SAT is thought to increase during adolescence and plateau in young adulthood in individuals of healthy, stable weight [29]. However, obese individuals have almost double the number of adipocytes in SAT compared to non-obese individuals. A number of studies have supported the idea that weight gain induces a new threshold of the adipocyte number that cannot be reversed even in the context of weight loss [29,62]. However, what regulates this observed hyperplasia under conditions of WAT expansion? To determine factors that contribute to hyperplasia in AT, a recent study identified TGFβ3, a protein that is released in the stromal vascular fraction of SAT, as a factor which stimulates the proliferation of adipocyte precursors and ultimately regulates the number of fat cells in vivo [63]. *Tgfb3* was initially identified as a candidate factor promoting proliferation by a transcriptomic analysis of human SAT which correlated genes with changes in the adipocyte number during weight gain. Further mechanistic studies revealed that attenuating TGFβ3 signaling actually blocked proliferation and instead produced adipocyte hypertrophy in SAT and glucose intolerance in rodent models. Though TGFβ3 can act through its target receptors to activate SMAD proteins [64], the precise mechanisms by which it contributes to proliferation of APCs is not known. However, TGFβ3 appears to be a critical component of the SVC fraction of AT that plays a role in the proliferation of preadipocytes that undergo differentiation in vivo, the prevention of adipocyte hypertrophy, and improved glucose tolerance at the systemic level.

## 4. Circadian Regulatory Mechanisms within Adipose Tissue

The circadian clock is an exquisitely regulated, 24 h time keeping system that exists in almost all cells of the body. The circadian clock regulates numerous physiological processes, ranging from the sleep/wake cycle, to cognition, and peripheral metabolism [65,66,67,68,69,70]. Large epidemiological studies have indicated that circadian disruption, as occurs in night and rotating shift workers, leads to metabolic disease, with waste adiposity, insulin resistance, and T2D being more prevalent compared to non-disrupted subjects, reviewed in [71,72]. Though its specific functions in different cell types within AT are still being discovered, a growing number of studies have revealed an important role for the circadian clock in AT function and organism-wide energy balance [73].

Like other types of cells, the mammalian adipocyte contains a key transcriptional feedback loop that is composed of two transcriptional activators, Circadian Locomotor Output Cycles Protein Kaput (CLOCK) and Aryl Hydrocarbon Receptor Nuclear Translocator Like (*Arntl*, aka *Bmal1*). Together, these transcriptional activators are necessary for driving the cellular and, ultimately, organism-wide rhythms of a circadian nature. Though the vast majority of genes have been shown to oscillate in some tissue under a specific set of environmental conditions [74], 5–20% of a tissue transcriptome is thought to undergo daily rhythms in a specific physiological context. CLOCK and BMAL1, both bHLH-PAS (basic helix–loop–helix; Per-Arnt-Single) proteins, activate transcription by binding to specific DNA elements, E-boxes, in the promoters of target genes, including their own negative regulators, the Period (*Per*) and Cryptochrome (*Cry*) genes. Like other tissues of the peripheral nervous system, AT is highly rhythmic, with similar phase oscillations for the core clock components (i.e., CLOCK, BMAL1, PERs and CRYs) in BAT, SAT, and VAT [75,76,77,78,79,80,81,82,83]. Prior genome-wide studies performed in mice have revealed that approximately 4% of genes in WAT undergo rhythmic variation, while 8% of transcripts in BAT show diurnal oscillation [81]. Studies of the primate baboon show similar oscillations in the AT transcriptome, with between approximately 200 and 3000 rhythmic transcripts depending on the fat depot [84]. Recent studies analyzing human SAT biopsies taken throughout a 24 h period from healthy subjects under constant routine conditions revealed over 800 rhythmic transcripts [75]. While the mRNAs of genes that are involved in transcriptional regulation are a large component of the morning-peaking transcript group, evening-peaking mRNAs are comprised of a significant number of genes that are involved in redox activity. Similar to the oscillations observed at the level of gene transcription in fat, some metabolites also undergo daily oscillations in fat [77]. Considering the murine circadian metabolome of AT, BAT shows more rhythmicity than WAT, though not all rhythmic metabolites in BAT and WAT under conditions of energy balance are shared under conditions of diet-induced obesity [77]. For example, long chain non-essential fatty acids (NEFAs) are highly rhythmic in BAT under conditions of energy balance; however, these metabolite oscillations are largely lost under conditions of diet-induced obesity.

**Figure 3:**
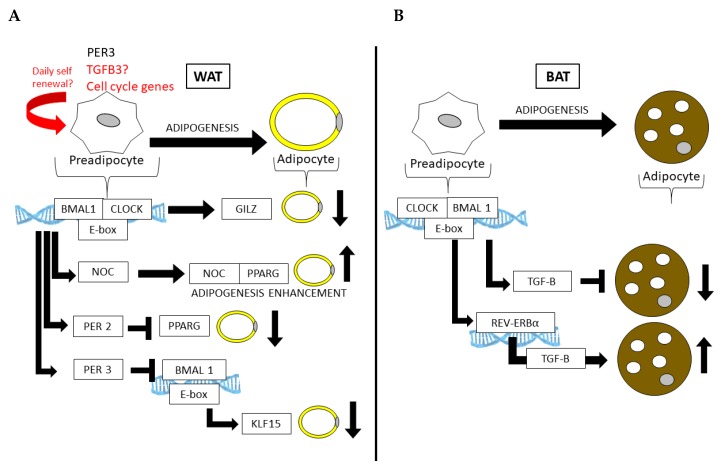
Core clock genes control adipogenesis in both (**A**) WAT and (**B**) BAT. In WAT, the core clock regulates genes that repress differentiation (*Gilz*) or promote the fate of a mature adipocyte (*Pparg*). Similarly, the CLOCK:BMAL1 regulation of *Per3* in APCs can prevent adipogenesis in WAT by the subsequent and direct downregulation *Klf15* by PER3 and BMAL1 at the *Klf15* locus.

Cyclic gene expression and metabolite abundance within AT is hardly surprising, considering that several of the normal activities of AT, such as lipolysis and lipogenesis, undergo 24 h rhythms. In fact, many of the key enzymes that are involved in lipolysis and lipogenesis are directly regulated by the CLOCK:BMAL1 transcriptional complex [79,85,86,87,88] (Figure 2A). Thus, lipolysis is highly rhythmic in WAT, resulting in daily fluctuations of free fatty acids (FA) and glycerol in the blood stream [88]. Circadian mutant mice have demonstrated the importance of the clock in lipolysis; circadian mutant mice have increased adiposity and dampened rhythms of free FA and glycerol release into the bloodstream [88], likely due to the fact that the normally rhythmic lipolytic genes, adipose triglyceride lipase (*Atgl*) and hormone sensitive lipase (*Hsl*), fail to oscillate in *Clock* mutant visceral WAT. (Figure 2B). Twenty-four hour physiological rhythms are also present in BAT (Figure 3). For example, BAT thermogenic capacity has been shown to be rhythmic, corresponding to the robust rhythms in REV-ERBα expression [83]. Specifically, mice exposed to cold at a *zeitgeber* time when REB-ERBα expression is low show a greatly improved tolerance to cold, based on REV-ERBs’ ability to transcriptionally repress *Ucp1* expression at this particular time of the 24 h cycle. In mice, FA uptake by BAT is also highly rhythmic, peaking at awakening and aligned with peaks in postprandial lipid handling [89]. Both mice and humans show a postprandial FA excursion rate following an equivalent meal that peaks during the early active phase, suggesting that other circadian mechanisms that are observed in mouse BAT are also present in human BAT.

Partial mechanisms by which the circadian clock functions in AT have been demonstrated in several mouse models with circadian defects [90,91], including the knockouts of or mutations in *Clock* and *Bmal1* [92] (summarized in Table 1). For example, *Clock* mutant mice, which express mutant CLOCK protein in all cells, become more obese and insulin-resistant on HFD compared to wild-type (WT) littermate controls [93]. The whole-body loss of BMAL1 leads to adipocyte hypertrophy and adiposity [78], as well as the adipocyte-specific loss of BMAL1 via the adipocyte protein 2 (*aP2*, or *Fabp4*) driver causes the defective circadian release of polyunsaturated FA and ultimately increases adiposity as a result of arrhythmic energy intake [79]. BMAL1 appears to have important functions in both WAT and BAT. While its loss increases adipogenesis and adipocyte hypertrophy in WAT, largely due to its inability to regulate Wnt pathway members that suppress adipogenesis [94], its loss leads to an increase in brown adipocyte cell fate, ultimately increasing thermogenic capacity in rodent models [95]. Adipogenesis can also be stimulated by the circadian deadenylase, Nocturnin (*Noc*), in a peroxisome proliferator-activated receptor gamma (PPARG)-dependent manner [96]. NOC plays a role in both WAT and BAT and is induced in BAT following cold exposure, where it is thought to play a role in long term metabolic adaptation [97]. Interestingly, PPARG also interacts with the circadian transcriptional repressor Period 2 (*Per2*), the loss of which results in enhanced PPARG recruitment to the target sites of adipogenic genes, promoting the induction of mostly brown adipogenic genes and increasing WAT oxidative capacity [86]. Another member of the Period genes, *Per3*, has recently been shown to robustly oscillate in APCs, where it too limits adipogenesis by repressing Krüppel-like factor 15 (*Klf15*) expression [98]. Furthermore, the CLOCK protein is thought to attenuate adipogenesis via the transcriptional regulation of glucocorticoid-induced leucine zipper (GILZ) [99]. Thus, numerous circadian clock and clock-controlled genes are directly involved in adipogenesis and AT function.

## 5. The Circadian Clock in Adipocyte Progenitors and Adipogenesis

While a number of studies have addressed aspects of circadian gene regulation or metabolism in whole AT, the number of studies focused on understanding clock function specifically in APCs is relatively limited in comparison. However, the current understanding of this AT cellular niche is that these cells express clock genes that are ultimately involved in adipogenesis by actions of the described traditional circadian transcriptional feedback loop. For example, fluorescence-activated cell sorting (FACS)-purified APCs (Sca1^+^, CD45^-^, and CD31^-^) from the SAT of m*Per2*^Luc^ mice [105] show robust rhythmicity in *Per2*-driven luciferase expression ex vivo [98]. Furthermore, the time-dependent harvesting of APCs from wild-type mice followed by FACS sorting has revealed rhythms in circadian clock gene expression, which are particularly robust at the *Bmal1* and *Per3* loci [98]. Interestingly, PER3 appears to be a strong regulator of adipogenesis in APCs, repressing the *Klf15* gene, which is known to promote adipogenesis in 3T3-L1 cells. This is consistent with earlier studies that described PER3 as an inhibitor of adipogenic cell fate [106]. Subsequent studies have revealed that the microRNA miR-181a promotes adipogenesis in primary patient-derived adipose stromal cells, as well as bone marrow stromal cells [107]. Interestingly, miR-181a increases adipogenesis by directly targeting Per3, ultimately resulting in the circadian de-repression of the adipogenic gene PPARγ. Thus, the period genes play a critical role in APC and adipogenesis, in part by controlling the transcriptional regulation of PPARγ [86,107]. Interestingly, there is evidence that the clock may be more robust in pre-adipocytes than in mature adipocytes. The culturing of pre-adipocytes from *Per2-luc* mice has revealed a greatly enhanced *Per2*-driven oscillation in *luc* expression compared to mature adipocytes following a serum shock [108]. As is the case for mouse ASCs, human adipose-derived stem cells have also been shown to have robust rhythms in circadian gene expression. Synchronized primary cultures of undifferentiated or adipocyte-differentiated ACSs reveal rhythms in the expression of several clock genes, though the differentiation state appears to make a contribution to the overall period length of the oscillation [109] (there is a strong likelihood that in vivo, circulating factors/*zeitgebers* would align these periods). Interestingly, in a recent study addressing rhythmic transcripts in human WAT [75], an interaction network analysis that was aimed at generating predicted molecular interactions to the 727 circadian genes revealed an interaction network for cell cycle/centrosome regulation factors. Cell cycle genes have been demonstrated as rhythmic in several tissues and, in some cases, are under the direct control of the circadian transcriptional regulators CLOCK and BMAL1 [110,111,112]. The interaction of cell cycle genes with circadian genes in human AT is interesting considering that the ability of mature adipocytes to undergo proliferation is thought to be greatly limited. However, this relationship might be better explained by the impact of cells within the stromal/vascular compartment of AT, which, under conditions of energy balance (as in the referenced study), contain the vast majority of APCs and can undergo cell division and adipogenesis to generate new adipocytes.

While the circadian clock appears to be present and active in APCs, could circadian factors ultimately help dictate their adipogenic cell fate? In fact, there is evidence that the eventual differentiation of APCs into mature adipocytes may depend in part on the circadian release of adipogenic hormones. Several adipogenic hormones, including ghrelin, insulin, glucocorticoids, and prolactin [113,114,115], have all been shown to oscillate in a 24 h fashion. By using an adipogenic hormone cocktail, one study revealed that rhythmic application of adipogenic hormone cocktail to cultured preadipocytes caused minimal differentiation, while constitutive application of adipogenic stimuli caused robust differentiation [116]. Further mechanistic studies revealed that in response to rhythmic adipogenic hormonal stimulation, CCAAT Enhancer Binding Protein Beta (CEBPB)-driven PPARG levels are pulsatile, seldom reaching a threshold of concentration within the preadipocyte to attain differentiation. Furthermore, these levels produce a flat and non-oscillatory circulating glucocorticoid level for 21 days in mice, causing a doubling in fat mass; however, dramatically increasing the amplitude of glucocorticoid rhythms (well over the endogenous amplitude) for a longer period of time (40 days), produced no such effect. That glucocorticoids normally oscillate in vivo is consistent with the relatively low level of preadipocyte differentiation and adipocyte turnover that is postulated to exist under homeostatic conditions in humans [117].

## 6. Potential Circadian Regulation of APC Proliferation and Exhaustion?

The idea that the circadian clock might serve as a daily signal for preadipocyte proliferation is intriguing, though not one that has been directly addressed in mammalian models to date. However, the concept is not unfathomable. Many cell cycle genes are capable of oscillating in a variety of cell types and tissues under specific physiological conditions [110], and the circadian clock has been shown to gate the cell cycle in a variety of cell types, as reviewed in [118]. Complicating the role of the circadian clock in AT expansion and function is that the rhythmicity of peripheral tissues, including AT, is heavily controlled by energy intake as it pertains to both the timing and quality [77,119,120,121,122,123,124]. Rhythmicity in feeding appears to be a critical regulator of 24 h rhythmicity in several peripheral tissues. For example, HFD can reprogram some peripheral clocks in a manner that impairs circadian coordination across tissues [77,120,125]. In contrast, restricted feeding, even under HFD, can prevent obesity and normalize circadian rhythms in peripheral tissues [122,123]. Diet has a strong effect on the circadian clock of AT [76,126], with rhythms in core clock genes changing in response to such nutrient stress [76,126] and a complete restructuring of the circadian metabolome, particularly in BAT [77]. HFD is also known to greatly augment the number of proliferating cells in the stromal vascular fraction of WAT [127]. Lineage tracing studies have validated that HFD contributes to WAT hyperplasia by the induction of PDGFRα+ cells, which can undergo white adipogenesis in vivo. Recent unpublished data from our groups suggest that the proliferation of APCs in healthy AT may be a diurnal activity, with peaks in proliferation at the end of the feeding cycle (light onset in nocturnal animals) and a trough at the end of the fasting phase. While HFD greatly increases the proliferative capacity in both WAT and VAT of rodents, diurnal control is completely lost. This raises the interesting hypothesis that the internal 24 h clock of adipose tissue may be a critical determinant of adipose tissue mass throughout adulthood under conditions of energy balance. Like other tissues, the clock of AT appears to be highly influenced by nutrient input, which may also have an indefinite effect on APC proliferation during adulthood and under continued nutrient stress. The effect of diet on APC proliferation raises the question as to whether under conditions of energy balance, daily rates of APC proliferation and differentiation are exquisitely controlled. It is tempting to speculate that obesity can lead to the over-activation and exhaustion of APCs by overriding the circadian clock in AT. Whether under conditions of diet-induced obesity, rates are elevated beyond the limit that is normally dictated by the circadian clock, be it by the loss of circadian adipogenic hormone secretion or by some additional mechanisms, has yet to be elucidated. Studies that address the cell-autonomous nature of the clock in APCs will be enlightening. As of yet, whether circadian functions within APCs depend strictly on an autonomous clock or rhythmic energy cures that function as *zeitgebers* has yet to be fully determined.

## 7. Implications of Adipose Stroma Overactivation

ASCs were originally discovered as the cells that have the characteristics of MSCs and were initially characterized in the bone marrow [128]. In culture, MSCs are multipolar or bipolar cells that are notably larger than other cell types and have a clearly defined nucleus and nucleoli. MSCs can typically easily adhere to and grow on uncoated plastic by secreting their own extracellular matrix (ECM) and making focal adhesions. Unlike epithelial and endothelial cells, MSCs do not make tight junctions with surrounding cells. ASCs are similar to bone marrow MSCs in their capacity for fibroblast colony-forming unit (CFU-F) formation and differentiation into mesodermal lineages (osteocytes, chondrocytes, and adipocytes) [129]. With the ease of isolation, inherent multipotency, and their trophic, angiogenic, and immunosuppressive functions, ASCs have been embraced as a cell type of choice in regenerative medicine [130]. Specifically, they have been shown to potentiate burn injury repair and lipotransfer [131]. ASCs have been also clinically tested for many other indications, although only autologous transplants of minimally manipulated cells have been approved [132]. As bone marrow MSCs, ASCs can be considered as the first aid kit of the body that responds to tissue damage [133]. Interestingly, we discovered that ASCs are mobilized from WAT in obesity and circulate systemically [134]. Based on that notion, it could be considered that having more AT with increased quantity of ASCs may be beneficial if they can engage in tissue repair. However, more and more pessimism has arisen from studies indicating that MSCs/ASCs lose their beneficial properties with age and obesity development, and they may, in fact, develop senescent/pathogenic properties [135]. While the transient expansion of ASCs may be part of healthy tissue response to nutritional or immunological cues, their chronic over-proliferation may have long-term metabolic repercussions. Specifically, there is accumulating evidence that the abnormal activation and excessive proliferation of ASCs can re-direct them from their normal role and have adverse physiological implications in patients with certain types of cancer and other fibrotic conditions.

## 8. Adipose Cell Engagement in Cancer

One of the initial indications that the activation of ASCs may underlie pathology is their mobilization in patients with colorectal, prostate, and breast carcinomas [136,137,138,139]. The progression of carcinomas is aggravated by obesity [140,141]. Our studies in mouse models have shown that excess WAT enhances cancer progression irrespective of diet [142] and that lipotransfer facilitation with ASC upon breast tumor resection is linked with a higher likelihood of cancer relapse [131]. Tumor stroma, composed of a mixture of various non-malignant cell types, is a foundation of carcinoma progression [143,144]. While the pool of tumor leukocytes is maintained by hematopoietic progenitors, cancer-associated fibroblasts (CAFs), a major component of the tumor stroma, are of mesenchymal origin [145,146,147]. This mixed population is partly derived from ASCs, which have been found to play an important role in tumor growth in animal models [148]. ASCs proliferate in obesity, migrate from WAT to tumors, and promote cancer progression, likely through mechanisms they activate for normal tissue repair [139,142,149]. An adipocyte-secreted matricellular protein, Secreted Protein Acidic and Cysteine Rich (SPARC), binding to β1 integrin on the ASC surface, is a molecular trigger of ASC mobilization [150]. Chemokines C-X-C Motif Chemokine Ligand (CXCL)1 and CXCL8 signaling upon their receptors, C-X-C Motif Chemokine Receptor (CXCR)1 and CXCR2, mediate the homing of ASC to tumors [138,151]. The poor survival of obese patients with prostate cancer has been linked to ASC trafficking from WAT to tumors, which is mediated by CXCL1 that is secreted from malignant epithelium and signaling on ASC via its receptor CXCR1 [138]. It was also found that the secretion of CXCL1 by tumors in the setting of obesity is induced by WAT leukocyte-derived interleukin IL-22 signaling through IL-22R on cancer cells [152].

Molecular mechanisms through which tumor-recruited ASCs promote cancer progression are being investigated. Chemokines that are secreted by ASCs recruit macrophages, which have a tumor promoting role [153]. However, ASCs within the tumor microenvironment also directly contribute to carcinoma progression. Intratumoral adipocytes, differentiating from infiltrating ASCs, stimulate the proliferation of adjacent cancer cells [142]. ASCs are a major source of the ECM, such as fibronectin, collagens, and matricellular proteoglycans such as decorin and SPARC crosslinking the ECM [154], which underlie tumor desmoplasia [155]. ASCs also secrete trophic factors that stimulate vascularization [142,156]. Moreover, some of the cancer-promoting effects of ASCs may be contact-dependent [157]. In a recent study, a role for CXCL12, an ASC-secreted chemokine, in prostate tumor growth and invasiveness was demonstrated [158]. In addition, roles for ASCs in therapy resistance [159] and metastasis [160,161] have also surfaced. As an illustration of metabolic symbiosis, ASCs increase nitric oxide synthesis in cancer cells, leading to their decreased mitochondrial respiration, increased glycolysis, and chemoresistance [162]. Bone marrow MSCs mute anti-tumor immune response through their effects on T-lymphocytes [163], and ASCs are likely to have similar immunosuppressive properties. Recently, we reported that ASCs induce the epithelial–mesenchymal transition (EMT), an important step in the progression of carcinomas to a chemoresistant and invasive phenotype [164].

In addition to non-differentiated ASCs, mature adipocytes have been discovered as a source of pathogenic stroma. Peritumoral WAT undergoing remodeling in cancer plays a particularly important role in patients with breast and prostate cancer [140]. In a recent study, we took advantage of a mouse lineage tracing model with reporter expression imprinted in cells that had passed through adipogenesis [56]. This allowed us to identify stromal cells that were derived from adipocytes (ex-adipocytes). Such adipocyte-derived fibroblasts were particularly frequently observed in WAT upon tumor grafting. Our unpublished data indicate that adipocytes, in addition to stromal cells, contribute to the population of CAFs during tumor progression. How the adipocyte-derived ASCs are different from other ASC populations remains to be established.

## 9. Adipose Cell Engagement in Fibrosis

Fibrosis is the condition of chronic pathological tissue scarring that results from excessive ECM deposition. In obesity, AT itself becomes fibrotic and underlies the state of chronic low-grade inflammation [23,165,166,167,168]. However, it is fibrosis in other organs, such is lungs, heart, kidneys and liver, which often leads to life-threatening conditions. Organ damage usually initiates as a wound healing response aimed at the restoration of the normal architecture and the recovery of function. Unfortunately, tissue repair in adults does often not come to completion in time and is complicated by infections and inflammatory conditions such as obesity and diabetes. When the protective accumulation of scar tissue does not become resolved, it gradually progresses to chronic sclerosis sand fibrosis. Obesity, hallmarked by the overgrowth of WAT, is associated with progression of a number of fibrotic conditions [167]. This includes chronic renal fibrosis [169] and liver cirrhosis [170]. Building evidence also points to the importance of AT remodeling for subcutaneous fibrosis development in patients with scleroderma [168].

The key cellular mediators of fibrosis are myofibroblasts, the contractile fibroblasts that express α-smooth muscle actin (αSMA). When activated, myofibroblasts secrete ECM molecules such as collagens and matricellular proteoglycans crosslinking the ECM [154]. In conditions of muscle injury and degeneration, the pivotal cell population has been defined fibro adipogenic progenitors (FAPs) that proliferate at the affected site and contribute to both fibrosis and fatty infiltrates [171,172]. Myofibroblasts/FAPs can be derived from several cell types [154,173], but their main assumed source is MSCs [174]. MSC-derived FAPs not only induce fibrosis but also depotentiate myogenic progenitors in conditions of muscle damage and dystrophy [175]. It is tempting to hypothesize that, like in cancer, the MSCs of WAT (ASCs), proliferating and mobilized in obesity, could be recruited as progenitors of FAPs/myofibroblasts driving fibrosis [3,56]. This is particularly likely in organs that are contacted by WAT, such as skin, muscle, and kidneys. Interestingly, there is a correlation between obesity and idiopathic pulmonary fibrosis, despite the lung not being surrounded by AT [176]. The systemic mobilization of ASCs in pathological settings could potentially explain the link of obesity with fibrosis in organs not directly interacting with AT [177].

**Figure 4:**
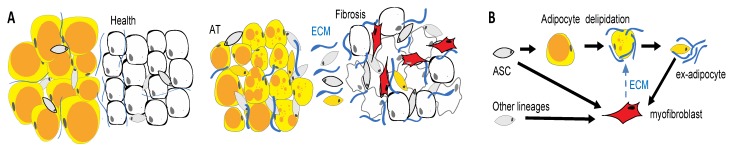
WAT cells as drivers of fibrosis. (**A**) Tissue remodeling in fibrosis. Pathogenic changes in the epithelium (white) induce the activation and proliferation of fibroblasts in adjacent adipose tissue (AT). (**B**) AT fibrosis is linked with loss of lipid droplets (orange) in adipocytes, which may convert into myofibroblasts that promote disease. Activated myofibroblasts (red) deposit an extracellular matrix (ECM). Adipose tissue is a key source of cells that convert into myofibroblasts secreting extracellular matrixes (ECMs) and driving fibrosis.

APCs undergo differentiation into adipocytes or fibroblasts depending on extracellular signaling [45,46,47]. The molecular mechanisms that direct adipose cell conversion to a pro-fibrotic phenotype are not completely understood. It has been shown that progenitor activation by TGF-beta/Smad signaling plays a role [46] in combination with inflammatory cytokines that are secreted by leukocytes [174]. The constitutive activation of PDGFR signaling has also been shown to induce fibrosis [45]. Recent single cell RNA sequencing studies have identified subpopulations of ASCs that are potentially predisposed to driving fibrosis. For example, the ASC of PDGFRb+ lineage in visceral mouse AT were classified into CD9/Ly6c-negative APCs and CD9/Ly6c-positive fibro-inflammatory precursors (FIPs) that suppress adipogenesis [47]. While these populations appear to be conserved in humans [178], relationships between them appear to be depot-specific and remain debated [61]. In addition to true progenitor cells of AT serving as pro-fibrotic progenitors, mature adipocytes undergoing lipolysis may contribute to fibrosis in pathological conditions [168,179]. We and others have shown that adipocytes can undergo complete de-differentiation into PDGFR+ ex-adipocyte stromal cells [56,180]. The loss of subcutaneous AT in patients with scleroderma is likely explained by this processes (Figure 4). Understanding the etiology and evolution of the distinct adipose cell populations in obesity and aging may provide key insights into their potential pathogenic roles in fibrotic conditions.

## 10. Implications of ASC Exhaustion for Adipose Tissue Dysfunction

**Figure 5:**
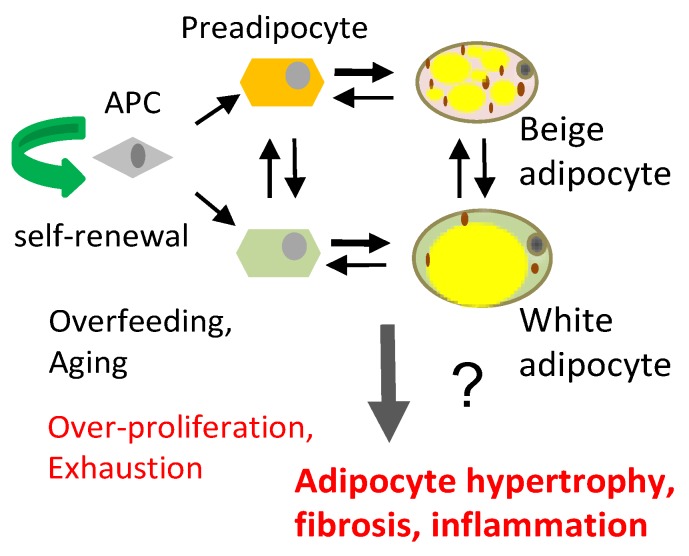
APCs differentiate into distinct lineages of preadipocytes: *Pdgfrβ-*predisposed to beige adipogenesis and *Pdgfrβ+* predisposed to white adipogenesis. The depletion of these lineages in different depots may have distinct effects on adipocyte number, size, and AT dysfunction, leading to fibrosis and inflammation.

Aging is associated with the development of life-threatening inflammatory/metabolic/degenerative diseases and cancer. Obesity, hallmarked by an overgrowth of WAT, predisposes one to the earlier development of these diseases and aggravates their severity. It has been shown that many manifestations of aging are triggered by the accumulation of senescent cells. Cellular senescence is an irreversible stress response that results in irreversible growth arrest [181]. Senescence is mediated by the expression of Ink4a, also known as p16, a protein that is encoded by the Ink4a/Arf locus [182], though senescence can also be achieved by activating the p53/p21 tumor suppressor pathway [181]. Senescent stromal cells, found in various organs, exert their pathogenic influence through and a pro-inflammatory secretory phenotype (Figure 5). This senescence-associated secretory phenotype (SASP) is characterized by the enhanced secretion of inflammatory cytokines, chemokines, growth factors, and proteases [183,184]. Senescence-inducing stimuli include tissue injury and remodeling, metabolic perturbations, radiation and, most importantly, the application of cytotoxic drugs [185]. The cellular decision between apoptosis and senescence is greatly influenced by the nature of the stress that is exerted. The presence of damaging agents such as cytotherapy may trigger a senescence-associated antiproliferative response rather than the activation of the caspase cascade that commits cells to apoptosis [186]. Cells that undergo senescence systemically exert various negative effects on the surrounding cells [181]. Recent studies have highlighted the role of senescent stromal cells in chronic inflammation and increased cancer aggressiveness [187]. Mouse models in which senescent cells are genetically tagged through the Ink4a promoter have been characterized [186,188]. The depletion of Ink4a-positive cells attenuated age-related organ deterioration, delayed tumorigenesis, and extended healthy lifespan in animal models [188].

WAT is a major site of senescent cell accumulation, which accounts for the chronic low-grade inflammation and the metabolic syndrome ensuing in obesity [184,189]. Importantly, the majority of senescent cells in WAT have been identified as ASCs [184]. It remains to be determined how the overproliferation and exhaustion of PDGFRα^high^/PDGFRβ^low^ and PDGFRαl^ow^/PDGFRβ^high^ APC populations [56] affects the pools of adipocytes in AT and tissue physiology (Figure 5). Previously, it has been reported that senescent cells have an increased expression of a PDGFRα ligand, PDGF-A [186]. Because the hyperactivation of PDGFRα signaling has been shown to induce fibrosis [45], this could be a part of the mechanism contributing to tissue dysfunction that is observed in aging. In humans, studies have shown that the accumulation of senescent APCs in SAT suppresses adipogenic differentiation [190] and that obesity intervention through long-term caloric restriction or bariatric surgery can delay APC senescence [190,191]. The question we face is what leads to the accumulation of senescent adipose cells and how this process can be suppressed. An important factor to consider is lifestyle, as high-fat diet is sufficient to accelerate cell senescence in WAT [192,193]. Oxidative damage, resulting from reactive oxygen species (ROS), is a major inducer of cell senescence [194]. DNA damage induced by ROS is likely to be an important trigger for senescence induction in mice, which, compared to humans, have relatively inefficient cellular mechanisms of protection against oxidative damage [195]. A particularly critical site of chromosomal damage by ROS is the telomeres, the protective caps at the ends of DNA strands [196]. Aging, obesity, and most metabolic and degenerative diseases are associated with telomere shortening in humans [197,198]. Within the context of AT, one theory of metabolic disease is that APC over-proliferation, aggravated by diet-induced obesity (DIO) may underlie their replicative senescence and exhaustion due to telomere attrition (Figure 1B). In support of this possibility, T2D is linked with more extensive telomere shortening observed in SAT of patients [199], which is possibly due to higher APC proliferation in SAT [200]. This theory has not been easy to study in the laboratory setting. Mice are not an ideal model to access the consequences of replicative senescence in WAT due to the prolonged ability of murine APC to repopulate AT with healthy small adipocytes [201]. This is due to the fact that while humans are born with telomeres in the 10–15 kb range, laboratory mice (C57BL/6) are born with telomeres of over 50 kb [202]. Thus, mice are more resistant to replicative senescence and stem cell depletion [195]. An example of this point is illustrated by Duchenne muscular dystrophy symptoms in mdx mice (lacking dystrophin), which develops much earlier in life when telomerase is genetically inactivated [203]. The generation of mouse models that simulate the telomere attrition that has been observed in humans would help to test the hypothesis that accelerated replicative APC exhaustion can lead to an unhealthy fat pad.

## 11. Adipose Cell Targeting

Though the roles for ASC in cancer is now well accepted, considering these cells as a prospective drug target has been barely explored. To date, there are no small molecule drugs that are available to selectively block the pathogenic effects of ASCs. We have used cytotoxin-conjugated peptides as experimental therapeutics to demonstrate the role of ASCs in cancer [52,204,205,206]. We have designed hunter-killer compounds that are composed of a peptide homing to ASCs [205] and of a pro-apoptotic domains. As we have reported, these compounds specifically induce apoptosis in ASCs ex vivo, as well as in a mouse model of diet-induced obesity [52]. These compounds can also be used for targeting AT-derived fibroblasts in tumors [206,207]. Our recent work has shown that the depletion of these adipose-derived CAFs modulates the level of ROS, EMT induction, invasiveness, and chemotherapy resistance in malignant cells [164]. Because clinical attempts to block CAFs have stirred controversy [147,208], more studies will be needed to consider these ASC-derived CAFs as potential therapy targets. More importantly, the repercussions of ASC depletion for AT health have to be considered. While ASC depletion enables a short-term metabolic benefit in mice [52], there may harmful long-term effect due to APC’s number limitation and inability to sustain fat pads with newly differentiated adipocytes. A potential solution to this problem may be in approaches to pharmacologically target senescent cells in AT without affecting functional APCs. Recent breakthroughs have demonstrated that senolytic compounds that clear senescent cells have promise in preventing or delaying life-threatening human disease and aging [184].

## 12. Discussion

In summary, accumulating evidence suggests that fibroblasts and senescent cells that are derived from ASC and adipocytes are the key drivers of fibrosis and inflammation associated with obesity and aging. The presence of a circadian clock in APCs and evidence that diet can control proliferation levels within this cellular niche of AT raise the question as to whether the daily regulation of APC proliferation and adipogenesis by our rhythmic energy intake pattern is a modulator of senescence and APC exhaustion over the lifetime of an organism. More experiments that directly address this question need to be performed. Regardless, if the generation of fibroblasts and senescent cells that are derived from ASCs do drive fibrosis and inflammation, targeting these AT-derived cells and/or their mechanisms of action, may be an effective approach to therapy against metabolic, degenerative, and fibrotic pathologies and cancer. It remains to be determined if nutritional intervention can suppress the replicative senescence of ASC and, hence, suppress or delay dysfunction of fat tissue and other organs in aging-associated diseases.

## Figures and Tables

**Table 1:** Effects of tissue-specific vs. whole body circadian gene knockouts on adipose tissue.

Gene	WAT	BAT
BMAL1 (whole body)	Increased adiposity but impaired adipogenesis Adipocyte hypertrophy	Increase in BAT mass and heightened cold tolerance [95]
BMAL1 (adipocyte-specific; adipocyte protein 2 [aP2] driver)	WAT expansion and loss of rhythmicity in polyunsaturated fatty acid release, driving arrhythmic eating [79]	Enhanced cold tolerance [100]
BMAL1 (brown adipocyte-specific, perivascular adipose tissue; Ucp1 driver)		Defective angiotensin production in PVAT. Reduced resting blood pressure, resulting in “superdipper” phenotype [101]
*ClockΔ19* mutant (whole body)	Increased mass and exaggerated WAT adipocyte hypertrophy on high fat diet [93] Increased adipogenesis in vivo and in cultured adipose-derived stem cells. Upregulation of adipogenic factors due to loss of transcription factor GILZ expression [99] Blunted lipolysis, resulting in loss of rhythmic glycerol and FA release [88]	
REV-ERBα (whole body)	More prone to diet-induced increases in fat mass Upregulation of βKlotho and FGF21 signaling in WAT [102]	Blocks neonatal BAT formation due to loss of brown lineage commitment [100] Improves cold tolerance in a zeitgeber-specific manner [83]
REV-ERBα/β (BAT-specific; Ucp1 driver)		Enhanced cold tolerance (via loss of suppression at Srebp1) [103]
PER2 (whole body)	Reduced fat mass, increased oxidative capacity in WAT Increase in adipogenesis-related genes (activation of PPARG targets) [86]	
PER3 (whole body)	Increased adipogenesis Increase proliferation of APCs in vivo (SAT) [98]	
Nocturnin (NOC) (whole body)	Protection from diet induced obesity, reduced visceral fat [104]	Altered long-term metabolic adaptation in to cold exposure [97]
